# Repair of Impacted Thermoplastic Composite Laminates Using Induction Welding

**DOI:** 10.3390/polym15153238

**Published:** 2023-07-29

**Authors:** Vedant Modi, Aswani Kumar Bandaru, Karthik Ramaswamy, Conor Kelly, Conor McCarthy, Tomas Flanagan, Ronan O’Higgins

**Affiliations:** 1 Bernal Institute, School of Engineering, University of Limerick, V94 T9PX Limerick, Ireland; v.modi@eirecomposites.com (V.M.);; 2 ÉireComposites, H91 Y923 Galway, Ireland; 3 Confirm Research Centre, University of Limerick, V94 T9PX Limerick, Ireland

**Keywords:** repair, carbon fibre reinforced thermoplastics, CF/PEEK, CF/PEKK, compression after impact, induction welding, fusion bonding

## Abstract

The lack of well-developed repair techniques limits the use of thermoplastic composites in commercial aircraft, although trends show increased adoption of composite materials. In this study, high-performance thermoplastic composites, viz., carbon fibre (CF) reinforced Polyetherketoneketone (PEKK) and Polyether ether ketone (PEEK), were subjected to low-velocity impact tests at 20 J. Post-impact, the damaged panels were repaired using an induction welder by applying two different methods: induction welding of a circular patch to the impacted area of the laminate (RT-1); and induction welding of the impacted laminates under the application of heat and pressure (RT-2). The panels were subjected to compression-after-impact and repair (CAI-R), and the results are compared with those from the compression-after-impact (CAI) tests. For CF/PEKK, the RT-1 and RT-2 resulted in a 13% and 7% higher strength, respectively, than the value for CAI. For CF/PEEK, the corresponding values for RT-1 and RT-2 were higher by 13% and 17%, respectively. Further analysis of the damage and repair techniques using ultrasonic C-scans and CAI-R tests indicated that induction welding can be used as a repair technique for industrial applications. The findings of this study are promising for use in aerospace and automotive applications.

## 1. Introduction

**Figure 1:**
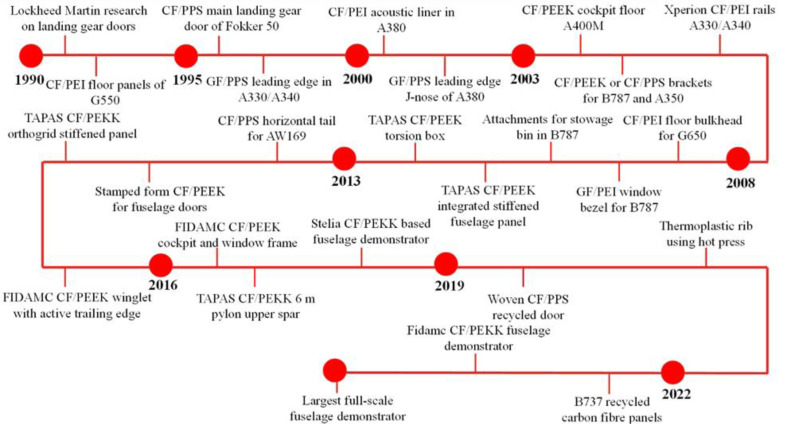
Thermoplastic structures in aviation.

Aircraft manufacturers are progressively introducing composite technologies, but continued R&D is critical to maintain aerostructure competitiveness. To meet demand, manufacturers need to improve production rates for composite components. Thermoplastic composites can be manufactured using various techniques and have been found to be suitable for producing large, complex geometries with relatively fast processing times. Figure 1 summarises the thermoplastic structures manufactured using thermoplastic composites. Despite the various advantages of thermoplastic composites, their acceptance for use in commercial aircraft could be faster. The vulnerability of composite commercial aircraft structures is a key concern [1]. While thermoset-based material systems have enhanced impact resistance, new efficient repair methodologies for damaged structures are still needed. Current certified composite primary structure repair is based on mechanical fastener methodologies, which are reliable but structurally inefficient. 

Adhesive bonding is a structurally more efficient method of joining structures. It offers good damping properties, protection from corrosion, lower weight and quicker assembly time compared with mechanical fastening. However, adhesive bonding requires surface treatment before bonding to improve adhesion. Sanding, grit-blasting, plasma and laser treatments [2,3,4,5,6] are the most commonly used surface preparation techniques. The current repair techniques for composite materials are labour-intensive and dependent on operator skill. Thermoplastics can be melted and formed, so they allow for another repair technique: fusion bonding [7]. The fusion bonding technique can eliminate the drawbacks of traditional joining techniques, generating optimised, reproducible, strong, and reliable joints with minimum surface preparation, cost, and cycle times. These techniques can be applied to high-performance thermoplastic composites with a high fibre volume fraction (>50%) and high processing temperature (>350 °C) [6]. Induction welding is an electromagnetic welding technique which has been studied for aerospace applications [8]. The induction welding process offers various advantages, including its short welding time, ease of automation and excellent bond quality. Furthermore, it allows for continuous welding and requires no foreign material at the joint interface. It represents an approximate 20% cost reduction and 15% weight reduction for the repair of composite materials relative to traditional techniques [9,10].

However, more research is required to develop efficient and reliable composite repair methods, enabling safer, lighter, and more efficient repair solutions for next-generation composite structures. The processing advantages offered by thermoplastics for high throughput and their inherent recyclability make them an increasingly attractive option for aircraft structures [11,12,13,14,15,16,17]. Aerostructures are susceptible to impact damage in-service, leading to catastrophic failures, thus, creating a need for the development of suitable repair techniques. As such, valid repair methodologies are required for thermoplastic composite structures when they enter service on commercial aircraft. 

Polyetheretherketone (PEEK) and Polyetherketoneketone (PEKK) are semi-crystalline polymers belonging to the poly-aryl-ether-ketone (PAEK) family. Relative to other engineering polymers, PEEK and PEKK have extraordinary mechanical, thermal, and chemical properties, making them suitable for aeronautical applications [17]. Relative to PEEK, PEKK has a higher glass transition (Tg) and melting temperature (Tm) due to a higher ketone ratio and better permeation and wear properties. Additionally, PEKK, as a polymer, offers an advantage over PEEK, as the former has a lower crystallisation rate.

Considerable work has been conducted based on the ability of thermoplastic composites to be remelted and reformed to evaluate repair methodologies. Table 1 summarises some of the repair work for thermoplastic composites from the literature. Rodgers and Mallon [18] and Miyake and Takenaka [19] assessed the repair of thermoplastic composites by employing induction heating. They reported a reduction in damage area and increased bending stiffness, respectively. Toyoda et al. [20] investigated the effect of patch repair on the bending of thermoplastic composites using ultrasonic welding. Their study concluded that the patch increases the bending stiffness with a limitation for large patches. Markus [21] employed laser beams to remove the area in a CF/PEEK laminate for scarf repair. Furthermore, induction heating was conducted to consolidate the patch on the laminate. Nijhuis [22] applied a patch on a CF/PEKK composite using a heat blanket and a heat gun for repair, concluding it to be a suitable repair technique for flat laminates. Zorer et al. [23] applied a hot-press patch on glass fibre-reinforced polypropylene to evaluate the impact response. The study reported a better impact response with lower energy absorbed for the patched laminate relative to the parent laminate. Bayazeid et al. [24] performed a repair methodology on CF/PEEK laminates employing induction heating. Using a custom drop test tower, the laminates were impacted at an energy of 10 J. Post impact, the laminates reported a damaged area and indent depth of 87 mm^2^ and 0.2 mm, respectively. Post repair, the damaged area and indent depth reported were 67 mm^2^ and 0.08 mm, respectively. However, a relatively small increase in the tensile strength was reported from 706 MPa post-impact to 730 MPa, post repair. More recently, Vreeken [25] performed ultrasonic and hot-press heating of impacted CF/PEEK laminates. Furthermore, the compressive residual strength post-repair was evaluated. However, none of the studies conducted assessed the difference between the effect of heating the composite and the application of a patch. Compression after impact tests were not performed to evaluate the restoration of strength, which is a critical parameter for repair.

In this study, CF/PEKK and CF/PEEK laminates were subjected to low-velocity impacts with an energy of 20 J. C-scans were conducted to evaluate the post-impact damage area. Induction welding was employed using two techniques: (a) welding a single-sided patch on the impacted laminates; and (b) heating the damaged area. Post-repair, C-scans were captured to assess the recovery of the damaged area. Furthermore, the repaired laminates were subjected to compression loading to evaluate the compression after impact and repair (CAI-R) strength.

## 2. Materials and Methods

### 2.1. Materials

Unidirectional CF/PEKK (TPUD_PEKK-HTS45) and CF/PEEK (TPUD_PEEK-HTS45) prepregs supplied by Teijin GmbH, Heinsberg, Germany, were used for this study. The parent and patch laminates were laid up by hand, and the specifications are provided in Table 2. The laminates were manufactured in an autoclave at temperatures of 365 °C and 375 °C for CF/PEKK and CF/PEEK prepregs, respectively. At the same time, the consolidation pressure and dwell duration were 600 kPa and 60 min for both materials. The temperature and pressure ramp-up and ramp-down rates were 3 °C/min and 50 kPa/min. The laminates for impact and external repair patches were extracted from larger composite panels using abrasive waterjet cutting.

### 2.2. Low-Velocity Impact Testing

Low-velocity impact (LVI) tests were performed on the parent laminates as per ASTM D7136 [26] using a custom-made drop test tower available at the University of Limerick, Limerick, Ireland. Typically, for LVI, the incident impact velocity ranges from 2 m/s to 5 m/s, resulting in impact energies of 10 J to 40 J [27,28,29,30]. However, 10 J resulted in insufficient damage area, and 40 J resulted in full penetration of the laminates. Therefore, for this study, the panels were impacted at 20 J. The test system had a 22 kN piezoelectric-load cell and a 4.3 kg hemispherical impactor. The impactor was released from a height of 0.5 m, resulting in an impact velocity of ~3 m/s and an impact energy of 20 J. The panels were rigidly clamped using four toggle clamps with rubber tips, and a pneumatic arm was employed to prevent the impactor from re-striking the impacted laminates. Simultaneously, Equation (1) from Ref. [26] was used to plot the load-time and energy-time curves for each impact event.

(1) $$v\left(t\right)=v_{i}+gt-\int_{0}^{t}\frac{F\left(t\right)}{m}dt$$

where t is the current time during impact (t = 0 at impact moment), F(t) is the measured impactor contact force at time *t*, and v(t) is the velocity of the impactor at time *t*. The actual velocity at impact, $v_{i}$ was established from the time the impactor took to pass between two laser sensors positioned 60 mm apart.

The displacement during impact, δ, was obtained from Equation (2):

(2) $$\delta \left(t\right)=\int_{0}^{t}v\left(t\right)dt=\delta_{i}+v_{i}t+\frac{gt^{2}}{2}-\int_{0}^{t}\left(\int_{0}^{t}\frac{F\left(t\right)}{m}dt\right)dt$$

where *δ_i_* = 0 is the initial displacement. 

The energy absorbed into the laminate is attained using the conservation of energy principle and is calculated using Equation (3):

(3) $$E_{a}\left(t\right)=\frac{m\left(v_{i}^{2}-v{\left(t\right)}^{2}\right)}{2}+mg\delta \left(t\right)$$

### 2.3. Repair Using Induction Welding

**Figure 2:**
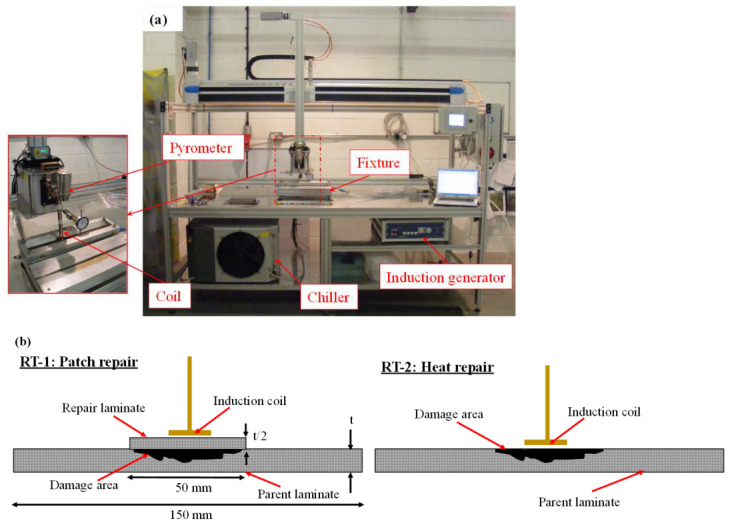
(**a**) Image of the induction welder at ÉireComposites, and (**b**) schematics for the two repair techniques.

The repair was carried out on an induction welding machine custom-built at ÉireComposites, Galway, Ireland. Figure 2a shows the components of the induction welding system: an induction power generator, a chiller, and a moving controller. A horseshoe-shaped induction coil with 18 mm × 32 mm dimensions was employed for heating. Furthermore, the temperature distribution on the upper surface of the composite laminate was measured using an infrared pyrometer (Thermometer laser M3 supplied by Micro Epsilon, Birkenhead, United Kingdom), as shown in Figure 2a.

Figure 2b presents the schematics of the two repair techniques (RT): for RT-1, a 50 mm diameter circular patch half as thick as the parent laminate was welded onto the impacted parent laminate; and for RT-2, the impacted area was heated by the induction coil and pressure was applied with a clamp. At a frequency and input power of 638 kHz and 1.9 kW, an upper surface temperature of 325 °C was recorded using the pyrometer. A pneumatically activated clamp applying a pressure of 3 bar was employed to hold the laminates in position. A repeat of three samples was performed for the RT-1 and RT-2 techniques for both the CF/PEEK and CF/PEKK laminates. 

The ultrasonic C-scanning technique (TecScan, Saint-Bruno-de-Montarville, QC, Canada) with a 20 kHz probe was used to quantify the internal damage in post-impact and post-repair laminates. The damaged area was computed using Image J^®^, Rockville, MD, USA. Furthermore, post impact and post-repair, CAI and CAI-R tests were performed on a 300 kN universal testing machine (Zwick Worcestershire, Worcester, UK) following ASTM D7137 [31]. 

## 3. Results and Discussion

### 3.1. Impact Test Results

**Figure 3:**
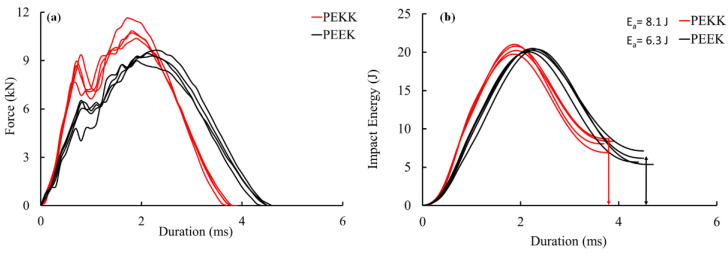
Impact response of CF/PEEK and CF/PEKK composites: (**a**) force and (**b**) energy.

Figure 3a,b present the force and energy absorption responses at 20 J for the CF/PEKK and CF/PEEK laminates. As the impactor penetrates the specimen, it stretches the target and creates an indent as the contact force increases. Sharp drops in the curve indicate damage initiation, which results in a sudden loss of stiffness in the contact area. Comparing the two laminates, CF/PEKK showed a 13% higher peak force than CF/PEEK. Furthermore, the threshold force for the CF/PEKK was 33% higher in comparison with CF/PEEK, suggesting damage initiated in the former at higher loads.

Furthermore, the difference in the thickness of the laminates influenced the impact force. High oscillations in the force response curve indicate damage. It can be seen in Figure 3a that at around 1 ms, the first drop in response indicates the initial failure of the laminate through matrix cracking and indentation. At this stage, the CF/PEKK laminate exhibited more decrease in force than the CF/PEEK laminate. This indicates that the CF/PEKK laminate absorbed more energy than the CF/PEEK laminate (Figure 3b). After 1 ms, the force increased to a peak value with fluctuations (indicating propagation of damage) and then decreased, indicating the complete failure of the laminate. The main concern is the contact duration, which depends on the peak force and energy absorption [32]. As mentioned in [32], the target absorbs the impactor’s energy through energy-absorbing mechanisms such as indentation, matrix cracking, fibre failure, contact duration and delamination [33]. Though the thickness of CF/PEEK (4.5 mm) was less than that of CF/PEKK (5.8 mm), the contact duration was high in the former laminate. This indicates the extent of damage within the CF/PEEK laminate, i.e., this laminate exhibited more internal damage with reductions in the impact force and the energy absorption. On average, the CF/PEEK laminates absorbed 6.3 J, compared with 8.1 J for the CF/PEKK laminates.

**Figure 4:**
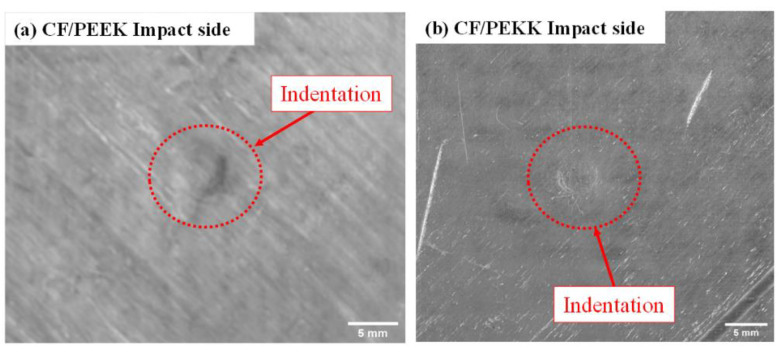

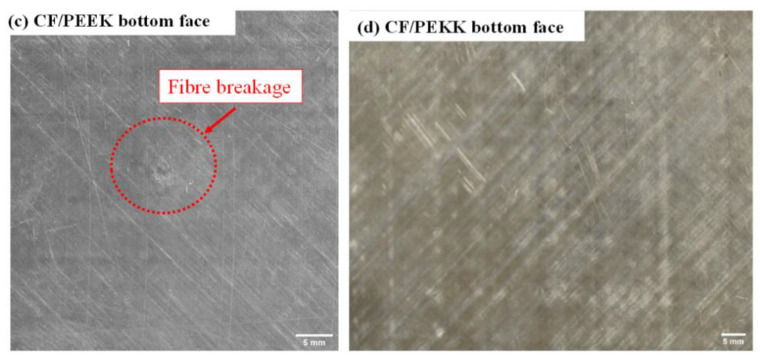
Optical micrographs of impact damage at 20 J.

Figure 4 shows the post-impact images of the impacted and opposite sides (bottom surfaces). Figure 4a,b show the indentation and matrix cracking on the impact face for CF/PEKK and CF/PEEK, respectively, at an impact energy of 20 J. The indentation on CF/PEKK was slightly more than that on the CF/PEEK laminate. In Figure 4c, CF/PEKK did not show any failure or bump on the back face, while CF/PEEK exhibited a small fibre protrusion on the bottom surface due to the onset of fibre breakage (Figure 4d). Typically, crack initiation is controlled by the fibre/matrix interaction which results in minor cracks followed by crack propagation and finally unstable crack progression at the fibre/matrix interface and crack kinks into the resin [34]. This indicates that CF/PEKK had delayed crack initiation at the fibre/matrix compared with CF/PEEK, resulting in higher peak force and damage resistance. Also, the damage resistance of CF/PEKK was due to the rigidity and ductility of the PEKK resin compared with the PEEK resin, and the PEKK resin exhibited high failure strains compared with the PEEK resin [17]. 

**Figure 5:**
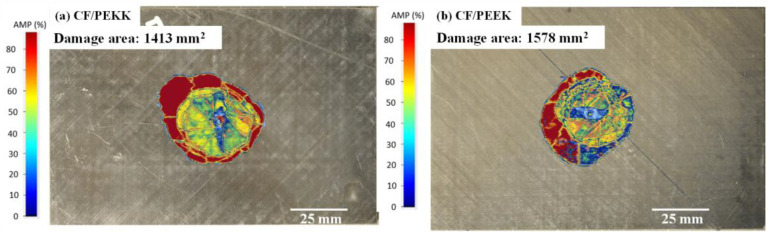
C-scan images of impact damage at 20 J.

Figure 5a,b show the ultrasonic C-scans and the damage footprint area for the CF/PEEK and CF/PEKK laminates. The C-scans show that the damage was initiated at the centre of the impact location, was circular, and propagated equally in all directions. Furthermore, the damaged area of the two material systems was similar. The damage observed in the C-scan for CF/PEKK was due to the indentation caused by the impactor, whereas CF/PEEK displayed a greater extent of delamination. The extent of delamination in CF/PEEK was due to the impactor’s longer contact duration, as mentioned in the above section. Similar results showed that the damaged area of CF/PEKK was 10% less than that of the CF/PEEK laminate, which can be attributed to the rigidity and ductility of PEKK resin over PEEK. According to Katunin et al. [35], the threshold for barely visible impact damage (BVID) is an indentation depth of less than 1 mm. In this study, the indentation depth for the CF/PEKK was less than 1 mm, while for CF/PEEK, it was more significant than 1 mm. 

### 3.2. Recovery after Impact

**Figure 6:**
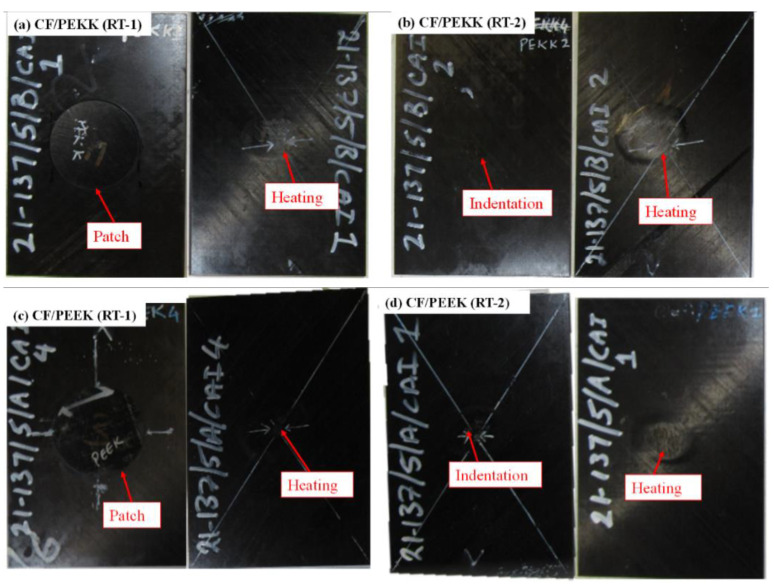
Comparison of two repair techniques.

Figure 6a,d show the laminates repaired by employing the induction welder. It can be seen in the above section that both the material systems showed a permanent indent on the impact face, and the CF/PEEK laminates exhibited fibre splitting and breakage at the bottom surface. During induction welding, three heating mechanisms occur: joule losses, junction heating and hysteresis heating [36]. Joule losses heat the carbon fibre when current is passed through them. The matrix must be dielectric and surrounded by carbon fibres for dielectric heating. Furthermore, the heat generated at the interface depends on the matrix properties and the frequency. For junction heating to occur, the fibres must be in contact with each other, and the heating depends on the contact resistance between the fibres [37]. The heating mechanisms resulted in a ring shape on the bottom surface of the laminate for RT-1 and RT-2. In contrast, for RT-1, applying pressure and the heating mechanism resulted in formation of a bond. Therefore, the repair technique, i.e., induction welding, resulted in local melting of the polymer, concealing the permanent indent on the impacted face (Figure 6a,d) and fibre splitting on the bottom face of the specimens.

**Figure 7:**
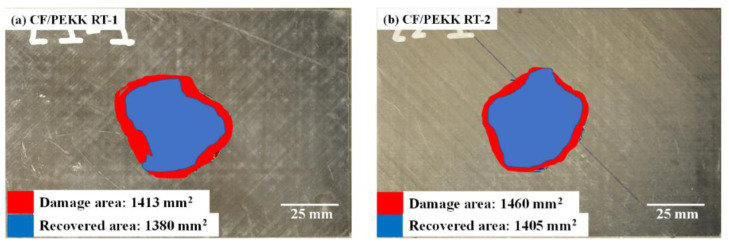

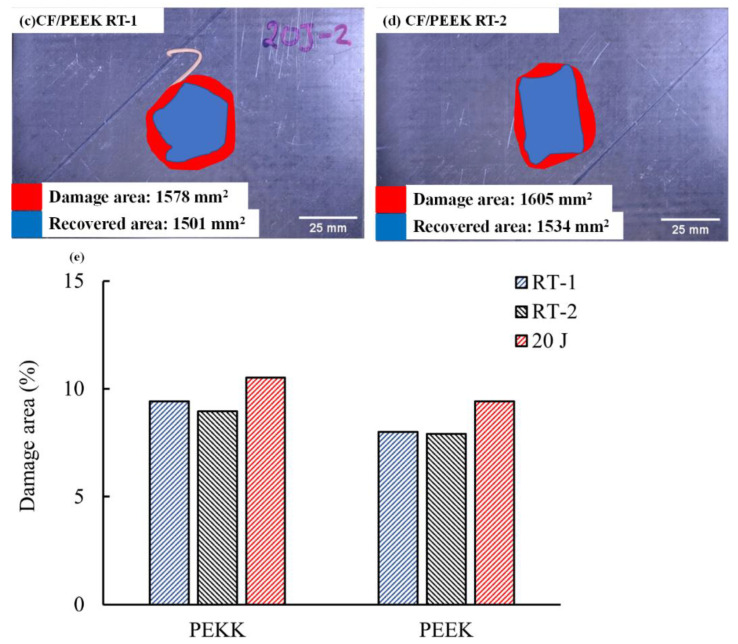
Ultrasonic scans for recovered damaged area (**a**,**b**) CF/PEKK (**c**,**d**) CF/PEKK and (**e**) Comparison of damage area before and after repair.

Post-repair, C-scans were conducted to determine the recovered damaged area. Figure 7 shows the representative damage area for the two material systems, post-impact and post-repair. In addition, Table 3 summarises the damage area post impact and repair for the two materials. RT-1 showed a ~3% and ~5% recovery of the damage area for CF/PEKK and CF/PEEK, respectively, at an impact of 20 J (Figure 7a,c). A ~4% recovery in the damaged area was observed for the RT-2 technique for the CF/PEEK and CF/PEKK laminates (Figure 7b,d). At 20 J impact, the CF/PEEK and CF/PEKK laminates had matrix cracks, delamination, and broken fibres. During induction welding, applying pressure and heat reduced the damaged area due to the reconsolidation of the matrix. However, the damage footprints observed in the C-scans post-repair were due to fibre breakages and caused a reduction in strength.

### 3.3. Compressive Strength Recovery

**Figure 8:**
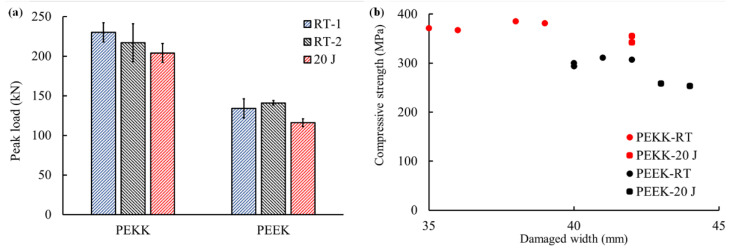
(**a**) Compressive load failure for different techniques. (**b**) Compressive strength to damage width.

Figure 8a,b show the compressive load to failure for the compression after impact (CAI), CAI-R tests and compressive strength. The CAI-R strength was higher for both material systems relative to CAI strength. The compressive strength presented in Figure 8b was calculated by dividing the peak compressive force by the cross-sectional area of the parent laminate, i.e., 100 mm × respective laminate thickness. The CAI-R strength was higher for both material systems relative to CAI strength. For CF/PEKK, RT-1 and RT-2 resulted in a 13% and 7% higher strength than the value for CAI. On the other hand, for CF/PEEK, the corresponding values for RT-1 and RT-2 were higher by 13% and 17%, respectively.

Figure 8b compares the CAI and CAI-R strength of the two material systems with the overall width of the damage measured during C-scans. The damage width for the two material systems varied from 36 mm to 44 mm for the repaired and impacted laminates. For both material systems, the CAI-R strength of the material increased with the reduction in damage width. The damage width is an important parameter when evaluating the compressive strength of a laminate. Under compression, the damage initiates in the vicinity of the damage width. Therefore, higher damage width lowers the compressive strength. 

**Figure 9:**
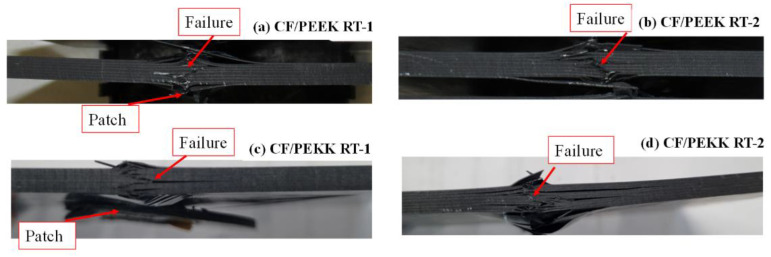
Failure cross-section of repaired composites after CAI tests.

Figure 9a,d show the final failure of the CF/PEKK and CF/PEEK samples for the two repair techniques post-CAI-R. Finally, as the failure in the RT-1 technique occurred away from the patch in the parent laminate, negligible strength improvements were realised, indicating no influence of the welding patch. It can be seen clearly that the extent of delamination in RT-2 technique samples was very high compared with the RT-1 samples. In both the CF/PEKK and CF/PEEK samples, the failure after the RT-1 technique was almost the same, which consisted of fibre failure and delamination near the impact zone. In the case of the RT-2 technique, the delamination was extended beyond the impact zone with buckling. Furthermore, comparing the CF/PEEK and CF/PEKK laminates, the former showed a brooming failure, whereas the latter showed a kink-band formation. The difference in the failure mechanisms is due to the higher ductility of the PEKK matrix, as reported in [17].

## 4. Conclusions

In this study, CF/PEEK and CF/PEKK laminates were impacted at 20 J using a drop tower. Induction welding was employed to repair the impacted laminates using two different techniques. In the RT-1 technique, a patch was welded on the parent laminate, whereas RT-2 involved heating the damaged area using an induction coil. Furthermore, C-scans were performed to measure the damaged area of the laminates post-impact and post-repair.

Induction welding resulted in a 3–4% reduction in damage area and a 7–17% increase in CAI-R strength for the laminates considered. Furthermore, applying a patch for repair using induction welding showed a minor improvement in strength compared with the heated laminate due to failure in the parent laminate. Therefore, when optimised, the heating of a composite laminate using an induction coil is a suitable repair technique. Induction heating is a non-contact method, with minimum consumables and no foreign material used at the welding interface, making it cost-effective and efficient. However, further experimental work evaluating the scarf or double patch repair for induction-repaired laminates is still required.

## Figures and Tables

**Table 1:** Summary of repair techniques using fusion bonding.

Author	Material	Repair Technique
Rodgers and Mallon [18]	CF/PEEK	Induction heating to assess damage area
Markus [21]	CF/PEEK	Induction heating to see if consolidation is possible
Nijhuis [22]	CF/PEEK	Heat blanket with pressure and vacuum to see if viable
Miyake and Takenaka [19]	CF/PEEK	Induction heating to assess the stiffness
Toyoda et al. [20]	CF/PEEK	Ultrasonic welding to assess bending stiffness
Zorer et al. [23]	GF/PP	Hot-press to evaluate impact response
Bayazeid et al. [24]	CF/PEEK	Induction heating to evaluate damage area and tensile strength
Vreeken [25]	CF/PPS	Ultrasonic heating to assess damage area and compressive strength

**Table 2:** Material properties for CF/PEEK and CF/PEKK.

Material Properties	CF/PEEK		CF/PEKK	
**Laminate type**	Parent Laminate	Patch	Parent Laminate	Patch
**Layup orientation**	[−45/0/45/90]_4s_	[−45/0/45/90]_2S_	[−45/0/45/90]_4s_	[−45/0/45/90]_2S_
**Laminate thickness**	4.5 mm	2.2 mm	5.8 mm	2.9 mm
**Laminate dimension**	150 × 100 mm^2^	∅50 mm	150 × 100 mm^2^	∅50 mm

**Table 3:** Summary of impact test and repair.

Material	Impact Energy (J)	Repair Technique	Damage Area (mm^2^)	Damage Width (mm)	Damage Indent (mm)	Compressive Strength (MPa)
CF/PEEK	20 ± 1	N/A	1578 ± 29	43 ± 2	3.2 ± 0.3	116 ± 5
CF/PEKK	20 ± 1	N/A	1413 ± 38	41 ± 1	1.9 ± 0.2	204 ± 6
CF/PEEK	20 ± 1	RT-1	1501 ± 31	41 ± 1	2.2 ± 0.4	134 ± 12
CF/PEKK	20 ± 1	RT-1	1380 ± 18	36 ± 1	1.4 ± 0.2	230 ± 8
CF/PEEK	20 ± 1	RT-2	1534 ± 26	39 ± 2	2.5 ± 0.3	141 ± 10
CF/PEKK	20 ± 1	RT-2	1405 ± 21	38 ± 1	1.7 ± 0.2	217 ± 22

## Data Availability

Data are contained within the article.
